# Use of latex microbeads for detection of *Plasmodium vivax* lactate dehydrogenase using flow cytometry

**DOI:** 10.1590/1414-431X2024e14114

**Published:** 2025-02-03

**Authors:** A.M.F. Franco, J.C. Glória, Y.O. Chaves, A.S. Ferreira, C.B.G. Teles, A.A.S. Balieiro, W.L.L. Neves, L.P. de Sousa, J.D.N. Costa, P.A. Nogueira, L.A.M. Mariúba

**Affiliations:** 1 Programa de Pós-Graduação em Biotecnologia, Universidade Federal do Amazonas, Manaus, AM, Brasil; 2 Instituto Leônidas e Maria Deane, Fundação Oswaldo Cruz, Manaus, AM, Brasil; 3 Programa de Pós-Graduação em Biologia da Interação Patógeno-Hospedeiro, Fundação Oswaldo Cruz, Manaus, AM, Brasil; 4 Plataforma de Bioensaios de Malária e Leishmaniose, Fundação Oswaldo Cruz, Unidade Rondônia, Porto Velho, RO, Brasil; 5 Programa de Pós-Graduação em Biodiversidade e Biotecnologia da Amazônia Legal - BIONORTE, Porto Velho, RO, Brasil; 6 Programa de Pós-Graduação em Biologia Experimental (PGBIOEXP), Fundação Universidade Federal de Rondônia/Fiocruz Rondônia, Porto Velho, RO, Brasil; 7 Fundação Hospitalar de Hematologia e Hemoterapia do Amazonas, HEMOAM, Manaus, AM, Brasil; 8 Laboratório de Pesquisa em Malária, Instituto Oswaldo Cruz, Fundação Oswaldo Cruz, Rio de Janeiro, RJ, Brasil; 9 Agência Estadual de Vigilância em Saúde de Rondônia, Porto Velho, RO, Brasil; 10 Programa de Pós-Graduação em Imunologia Básica e Aplicada, Instituto de Ciências Biológicas, Universidade Federal do Amazonas, Manaus, AM, Brasil

**Keywords:** Flow cytometry, Microbeads, Malaria, Plasmodium, Bead assay

## Abstract

Malaria is a parasitic disease of great relevance in global public health. The development of new sensitive and specific diagnostic high-throughput methods remains a challenge in the eradication of this disease. In this study, we developed a flow cytometry test using latex microbeads and polyclonal antibodies obtained from rabbits and mice for the detection of the *P. vivax* lactate dehydrogenase (PvLDH) antigen. We processed 50 samples from Brazilian patients diagnosed with malaria caused by *P. vivax* and 40 samples from healthy individuals. The assay presented sensitivity of 64%, specificity of 97%, a positive predictive value of 97%, and a negative predictive value of 57% when analyzed using the fluorescent labeling method. Using the mean fluorescence intensity (MFI) analysis method, the sensitivity was 53%, specificity was 89%, the positive predictive value was 95%, and the negative predictive value was 33%. In both methods of analysis, we observed significant statistical differences between the analyzed groups (P-value <0.0001). A high correlation (0.60) between the two methods and a low correlation between PvLDH concentration and parasite density was found. The test was able to detect the PvLDH protein with high specificity, but its sensitivity should be improved. More promising results were observed when the samples were analyzed according to the percentage of fluorescent labeling. Improvement of this assay would enable its application as a serological test for the detection of asymptomatic patients and for the validation of rapid diagnostic tests.

## Introduction

Malaria remains a serious global public health problem, with high numbers of cases and a high mortality rate. In 2021, approximately 247 million cases of malaria and about 619,000 deaths related to the disease were recorded worldwide (1). In Brazil, 139,112 cases and 50 deaths were recorded in that year (2). Early diagnosis is one of the control measures that is available for reducing the impact of the disease since it allows rapid treatment of the patient and thus disrupts the life cycle of the parasite (3).

In areas where malaria is endemic, the diagnosis of parasitic infections in asymptomatic patients is still a challenge in irradicating the disease, since these individuals contribute to the perpetuation of the chain of transmission (4). Therefore, there is a continuous need for the development and improvement of new diagnostic methods with high sensitivity and specificity to eliminate this disease.

Immunoassays using bead-based flow cytometry, whether simplex or multiplex, have valuable advantages in terms of cost, reproducibility, sensitivity, and specificity of serological tests, especially in assays for the simultaneous detection of different antigens (5,6). This diagnostic alternative has been used for the identification and confirmation of malarial antigens, as demonstrated by Rogier et al. (7,8), who developed a flow cytometry test based on polystyrene and magnetic microbeads using commercial monoclonal antibodies for the detection of *Plasmodium falciparum* HRP2 protein and *Plasmodium vivax* LDH using the Luminex^®^ platform (Thermo Fisher Scientific, USA).

In this study, we describe the development of a diagnostic method for malaria using flow cytometry based on latex beads for the detection of *P. vivax* lactate dehydrogenase (*Pv*LDH) protein, a well-established infection marker used in commercial tests for the detection of vivax malaria (9) employing polyclonal anti-*Pv*LDH antibodies obtained from rabbits and mice, which were produced against recombinant proteins of this malaria infection biomarker. As a result, we developed an alternative approach for malaria diagnosis caused by *P. vivax*, with the potential to be improved to enable sensitive and specific diagnosis using polyclonal antibodies.

## Material and Methods

### Sample collection

The samples were collected at the Centro de Pesquisa em Medicina Tropical (CEPEM/SESAU), Porto Velho, Rondônia, under approval of the institution’s ethics committee (CAAE: 28823719.4.1001.0005). Patients with a positive diagnosis by microscopy for malaria caused by *Plasmodium vivax* were invited to participate in the study and signed the informed consent form of the approved project. Patients of both sexes aged between 18 and 70 years with no signs of severe malaria were included, with thick blood smear positive for *P*. *vivax*, and parasitemia of ≥100 and <100,000 asexual parasites/µL. Samples from patients with a mixed malaria infection, history of malaria in the last 6 months, pregnant patients, hypersensitivity to antimalarial drugs, and use of antimalarials in the last 60 days were not included. The control group included samples from individuals who did not live in risk areas and had no symptoms of malaria in the last 6 months obtained at the Instituto Leônidas e Maria Deane (ILMD - FIOCRUZ AMAZÔNIA). Cytometry tests were performed on 50 vivax-positive malaria samples and on 40 samples from healthy subjects.

### Acquisition of polyclonal antibodies

The rabbit and mouse polyclonal anti-*Pv*LDH antibodies used in this study were obtained by our group in a previous study by Sousa et al. (10). Therefore, in this study, we used rabbit anti-*Pv*LDH PAbs as capture antibodies for the recognition of a *Pv*LDH (fragment 1-43 aa). *Pv*LDH 35-305aa PAbs obtained in mice were used as primary antibodies for recognition of *Pv*LDH almost in their entirety.

### Preparation of beads

The activation of latex beads (CML Latex Beads, 4% w/v, 4 µm; Invitrogen^®^, USA) was performed when placed in the presence of 0.57 µg/µL EDAC (1-ethyl-3-(3′-dimethylaminopropyl) carbodiimide) (Sigma Aldrich™, USA) and 2 mM/µL NHS (n-hydro-xysulfosuccinimide) (Sigma Aldrich™) diluted in PBS-1x and incubated for 3 h at 22°C with shaking at 950 rpm. Polyclonal anti-*Pv*LDH 1-43aa antibodies were then added at a concentration of 1 µg antibody to 1 µL beads. This mixture was lightly shaken for 16 h to allow covalent binding to the beads.

Coupled beads were blocked with blocking buffer (PBS-1x with 5% BSA) under light shaking for 2 h to avoid binding of nonspecific proteins. After the blocking period, the solution was washed twice with filtered PBS-1x + 0.5% BSA wash buffer and stored at 4°C until use.

### Cytometry tests

Blood samples were prepared by diluting 100 µL in 100 µL PBS-1x pH 7.4 + 0.1% Triton and incubated for 10 min. Samples were then incubated overnight in 100 µL of beads coupled under shaking at 950 rpm at 4°C. Then, a washing cycle was performed, as previously described. Subsequently, 100 µL of the primary antibody anti-*Pv*LDH 35-305aa from mice was added at the same concentration as the capture antibody. This was incubated for 1 h under light shaking at 22°C, followed by 1 wash cycle. The next step was incubation for 30 min with 100 µL of Alexa 488 secondary anti-mouse antibody (Thermo Fisher Scientific™, USA) diluted 1:2,000. Then, 1 washing cycle was performed, followed by resuspension in filtered PBS-1x, and reading was performed in a flow cytometer (FACSCanto II™, BD Biosciences™, USA). For data analysis, 50,000 events were obtained.

Data were analyzed using the FlowJo program (version 10, USA) and took into account size (FSC-A) and complexity (SSC-A) (Supplementary Figure S1), in addition to fluorescence percentage analysis and mean fluorescence intensity (MFI) (Supplementary Figure S2) to determine positive events in the samples that were above the “cut off” relative to the negative control samples.

### Statistical analysis

All analyses were performed with R software (version 4.0.2), R studio (version 1.1.4), and GraphPad Prism (version 9). The cut-off point was calculated using the mean of the negative controls plus two times the standard deviation. The Mann-Whitney test was performed for the measurement parameters, with P<0.0001 considered to be statistically significant.

## Results

The immobilization of antibodies was evaluated by analyzing the percentage of latex bead coupling. Our results showed an average of 96% of coupled microbeads (Supplementary Figure S1A). Different populations of microbeads with different sizes and complexities were observed, all of which were selected for single-cell analysis and detection of fluorescence (Supplementary Figure S1A-C).

After standardization of antibody coupling with the latex beads, the determination of anti-*Pv*LDH recognition sensitivity in biological samples was performed, which made it possible to distinguish the difference between samples with the presence of plasma levels of *Pv*LDH protein and those without the presence of plasma levels of *Pv*LDH in the detection systems (Supplementary Figure S2A-D).

**Figure 1:**
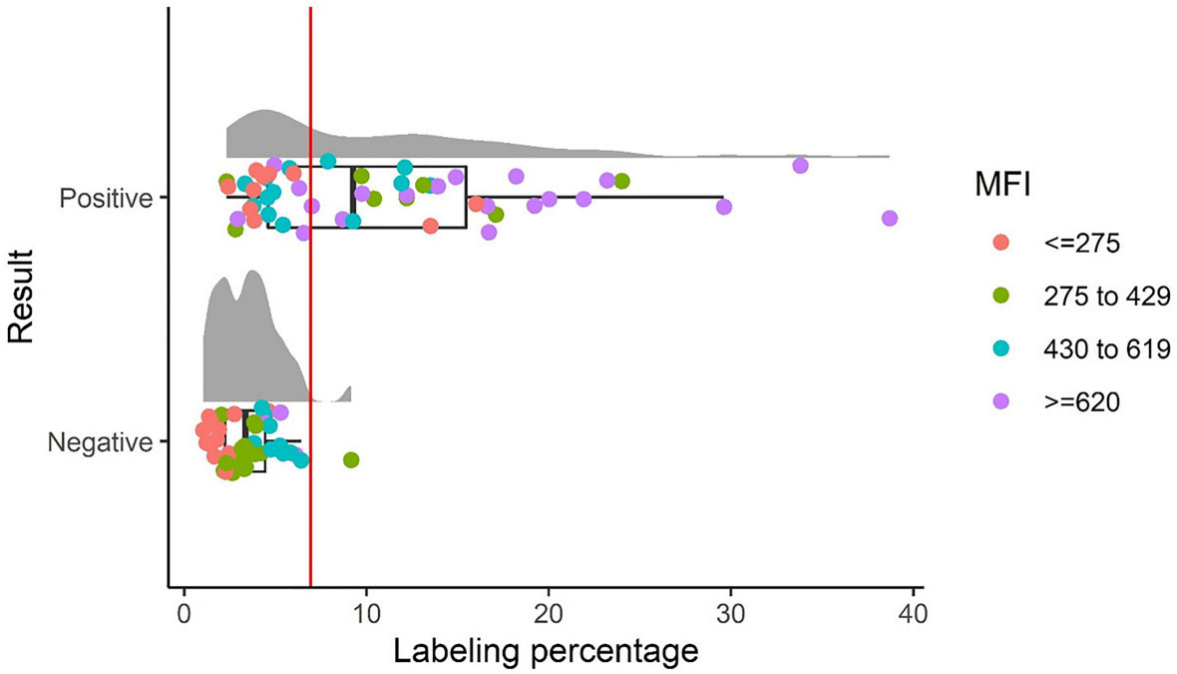
Raincloud graph for fluorescence percentage analysis of the number of anti-*P. vivax* lactate dehydrogenase (PvLDH) events. The graph shows the percentage of identified events in the coupling of the anti-PvLDH antibodies to latex microbeads for the recognition of the LDH antigen present in blood samples. On the Y-axis, the two groups of are separated into positive (n=50) and negative (n=40) samples, and on the X-axis, the percentage of fluorescent labeling is shown. The vertical red line at 7% fluorescent labeling represents the cut-off line. The mean fluorescence intensity (MFI) of the samples is separated into quartiles, represented in red dots for MFI of ≤275, green dots for MFI of 275 to 429, blue dots for MFI of 430 to 619, and lilac dots for MFI of ≥620. When compared, the two groups presented a statistical difference (P<0.0001, Mann-Whitney test).

An analysis based on the percentage and mean fluorescence intensity was performed to determine which parameter determines better sensitivity and specificity. The analysis of the samples based on the percentage of fluorescent labeling (PFL) of the detected events showed a statistically significant difference between the groups analyzed (P<0.0001, Figure 1), sensitivity of 64%, specificity of 97%, a positive predictive value 97%, and a negative predictive value 57%, with values being considered positive when above 7%.

**Figure 2:**
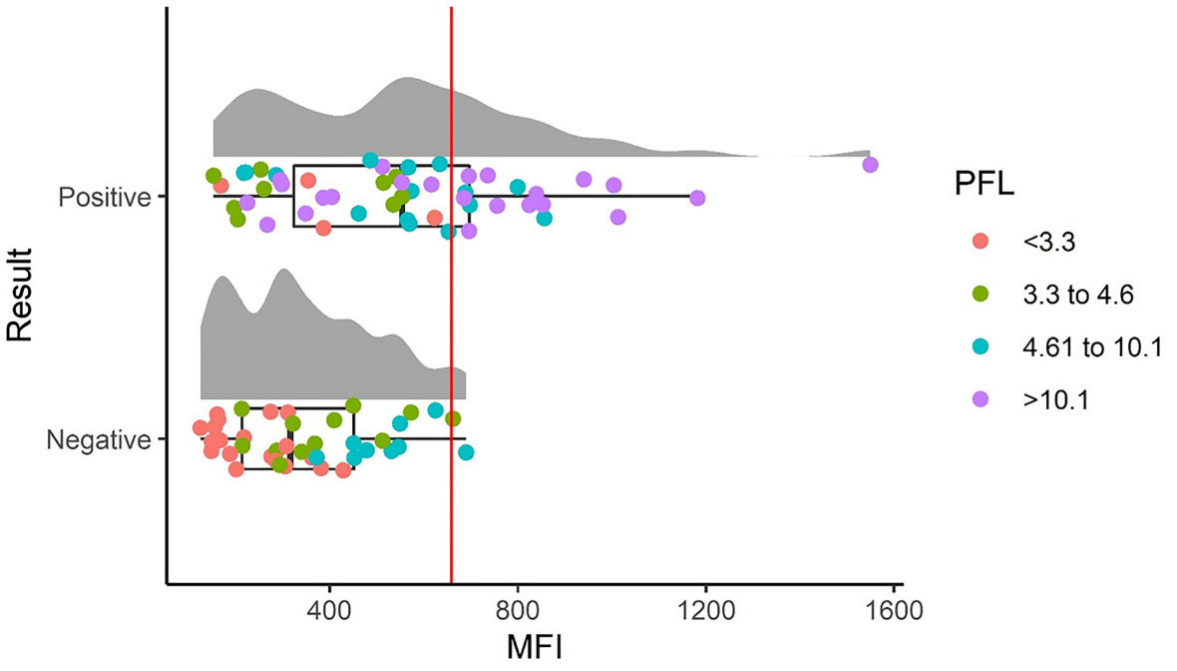
Raincloud graph for analysis of mean fluorescence intensity (MFI). The results showed the MFI identified in the coupling of the anti-*P. vivax* lactate dehydrogenase (PvLDH) antibody to the latex microbeads for the recognition of the LDH antigen present in the blood samples. On the Y-axis, the two groups are separated into positive (n=50) and negative (n=40) samples, and on the X-axis, the mean fluorescence intensity is shown. The vertical red line at MFI 659 represents the cut-off line. The percentage of fluorescent labeling (PFL) of the samples is separated into quartiles, represented by red dots for ≤3.3%, green dots for 3.3 to 4.6%, blue dots for 4.61 to 10.1%, and lilac dots for ≥10.1%. When compared, the two groups presented a statistical difference (P<0.0001, Mann-Whitney test).

**Figure 3:**
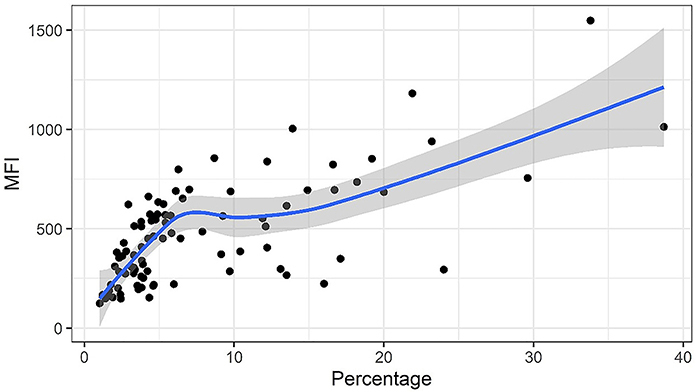
Graphic representation of the expected values for the relationship between mean fluorescence intensity (MFI) and the fluorescence labeling percentage. On the Y-axis, the MFI data are represented, and on the X-axis the percentages of fluorescent labeling are shown. The black dots represent the samples. The gray band represents the standard error and the blue line the expected value.

Similarly, MFI analysis showed a statistically significant difference between the groups analyzed (P<0.0001, Figure 2). The sensitivity of the assay was 53%, the specificity was 89%, the positive predictive value 95%, and the negative predictive value 33%, with MFI values above 659 considered positive. In addition, a high correlation (0.60) was observed between the two analysis methods (Figure 3), with no correlation between these and the parasitic density present in the tested samples. Additionally, the receiver operating characteristic (ROC) curve presented an area under the curve (AUC) of 0.74 (MFI, SD=0.05, P<0.0001) and 0.86 (PFL, SD=0.03, P<0.0001), both of which are considered acceptable (Figure S3), with the DeLong test result of -2.06 (P-value 0.04). A summary of the results is presented in Supplementary Table S1.

## Discussion

Immunoassays in bead-based flow cytometry have been successfully used in a wide variety of applications, such as early detection of SARS-CoV-2 (11), early detection of simultaneous biomarkers for several types of cancers (12,13), and identification of extracellular vesicles (14). For malaria antigen diagnosis, it is a promising tool since it has high sensitivity and specificity and has been successfully applied for the validation of rapid tests, commonly used in areas of difficult access and for epidemiological studies around the world (15).

In the present study, the use of polyclonal capture and detection antibodies was appropriate for serological tests, as demonstrated by Sousa et al. (10) when using the same polyclonal antibodies in an ELISA test for the detection of vivax malaria. In that trial, a positive predictive value (PPV) of 99% and a negative predictive value (NPV) of 45% were achieved. The present study found higher values for NPV (64%) and a similar result for PPV (97%).

Jang et al. (16) developed a multiplex test for the simultaneous detection of four malarial antigens (specific and pan-specific): HRP2, *Pf*LDH, *Pv*LDH, and *P. vivax* lactate dehydrogenase (pLDH). The assay had a specificity of 99% and a lower sensitivity in all evaluated antigens, the lowest value being for *Pv*LDH (46.1%). Similarly, our test showed a high specificity (97%) and an even higher sensitivity than that reported by Jang et al. (16) (64%) when using PFL. A low sensitivity to this antigen is also reported in rapid diagnostic tests for *P. vivax* identification (17), being a challenge for new diagnostic approaches to increase the sensitivity of this biomarker. Rogier et al. (8) reported a high specificity (100%) and sensitivity (91.7%) in the detection of *Pv*LDH by a multiplex flow cytometry test, but cross-reactivity was observed in some samples of patients with malaria caused by *P. falciparum*. Martiáãez-Vendrell et al. (18) described another targeted cytometric bead array assay to detect the pLDH antigen, a pan-specific biomarker, showing high specificity and sensitivity. Nonetheless, the assays achieved lower sensitivity when applied to *P. vivax* samples, and the authors reported greater sensitivity for detection in patients with *P. falciparum,* demonstrating once again the great difficulty in the specific detection of *Pv*LDH.

The low correlation between *Pv*LDH concentration and parasite density observed in this study has already been reported by Markwalter et al. (19) who evaluated an assay for pLDH detection using microbead-associated ELISA. It is believed that biological factors, such as the stage of the parasite’s life cycle, can potentially affect antigen expression, causing the low strength of the observed correlation (20). The observed positive correlation between the values obtained by the MFI and the percentage of fluorescent labeling was expected and is probably due to the fact that they have the same detection origin. However, when analyzing the samples individually, a dispersion in the distribution of these values is observed, since there are samples with a high PFL and low MFI and *vice versa*. Further work will seek to improve the method and the correlations described.

Despite the high specificity of the test described here, future studies should seek to increase the sensitivity of the assay. We believe that the high level of impurities in the samples, even after long washes, hindered the achievement of better results. Future evaluation of the use of other types of microbeads, such as polystyrene, polyacrylamide, glass, magnetized or not, may be a way to improve the performance of the test. Such modifications can increase the purity of the sample to be analyzed and allow unique populations of microbeads to be obtained for analysis and positively influence the correlation of fluorescence analyses with the parasite density present in patients.

The prototype flow cytometry assay using latex microbeads developed in this study thus proved promising for the detection of malaria caused by *P. vivax*. Rogier et al. (7,8) used monoclonal antibodies and magnetic beads, which made the technique costly to execute. Polyclonal antibodies have a greater risk of cross-reactivity with other proteins, but when produced against specific regions of the target antigen associated with isolation steps, they can become a less expensive alternative for producing inputs for diagnostic tests.

The work developed here used low-cost latex beads, which were easy to handle and had an adequate size for flow cytometry analysis. Furthermore, this study represents an advance over the analysis of polyclonal antibodies developed by Sousa et al. (10), since flow cytometry offers greater accuracy compared to ELISA. Future tests may be performed to improve the sensitivity of the assay or even apply the method described here for the detection of other diseases. In the future, these assays may compose a multiplex test aimed at the simultaneous diagnosis of several diseases.

## Supplementary Material

Click to view [pdf].
