# Supplementary material for: Use of latex microbeads for detection of Plasmodium vivax lactate dehydrogenase using flow cytometry
Source: Braz J Med Biol Res. 2025 Feb 3;58:e14114. doi: 10.1590/1414-431X2024e14114 (PMC11793140; doi:10.1590/1414-431X2024e14114)
Supplement: Supplementary file 1 [file 1414-431X-bjmbr-58-e14114-suppl.pdf]

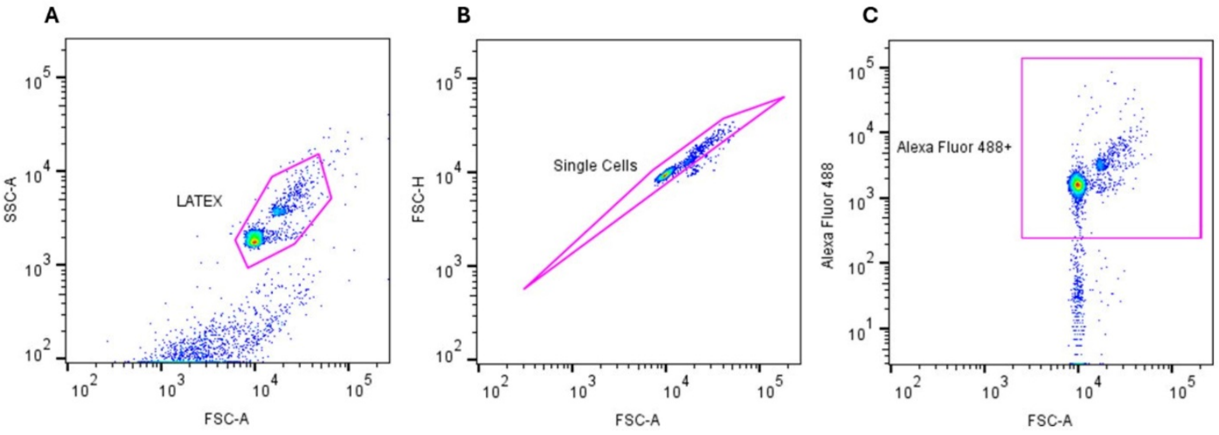

Figure S1. Determination of antibody coupling in latex beads using flow cytometry. **A**, Morphometric analysis of size (FSC-A) and complexity (SSC-A) of the latex particles; **B**, single cell analysis; **C**, dot plot of latex particles with coupled antibodies.

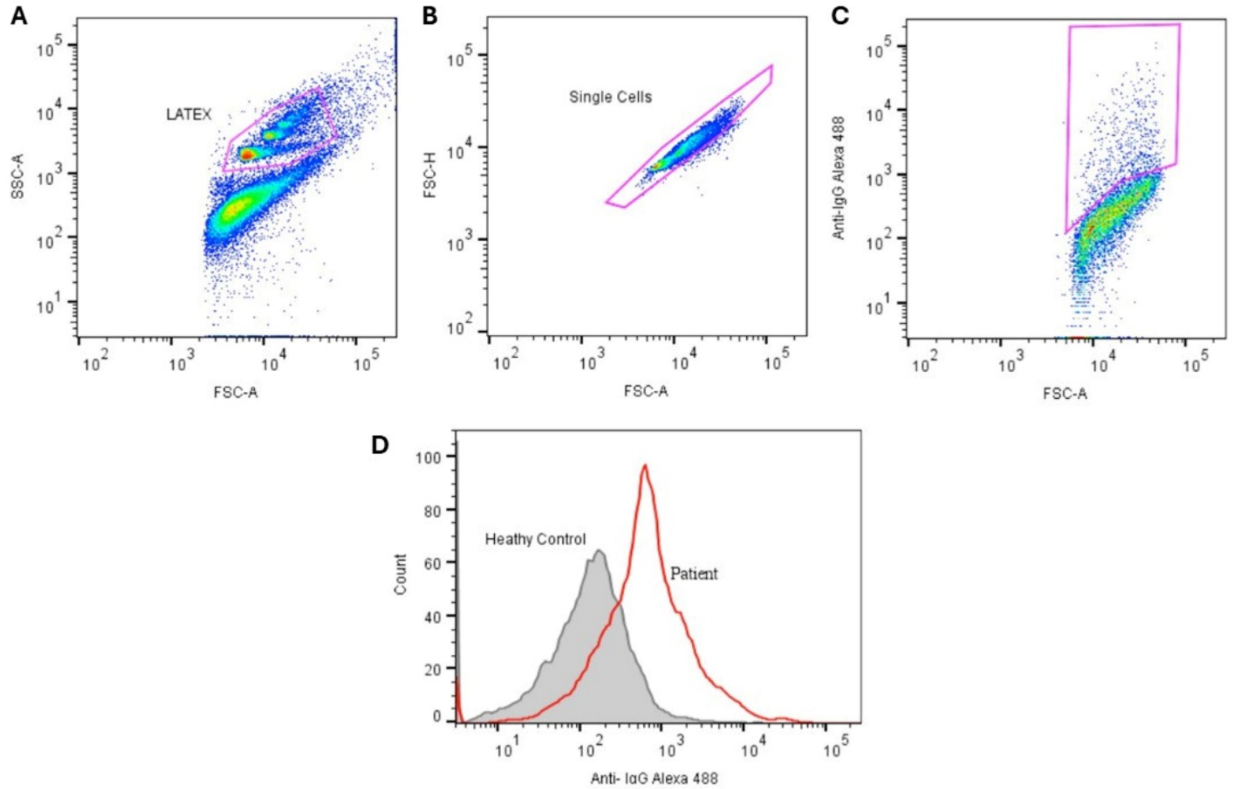

Figure S2. Determination of anti-*P. vivax* lactate dehydrogenase (PvLDH) recognition sensitivity using flow cytometry. **A**, Morphometric analysis of size (FSC-A) and complexity (SSC-A) of latex particles; **B**, single cell analysis; **C**, dot plot of antiPvLDH IgG in latex particles; **D**, determination of mean fluorescence intensity between the control group and the patient group.

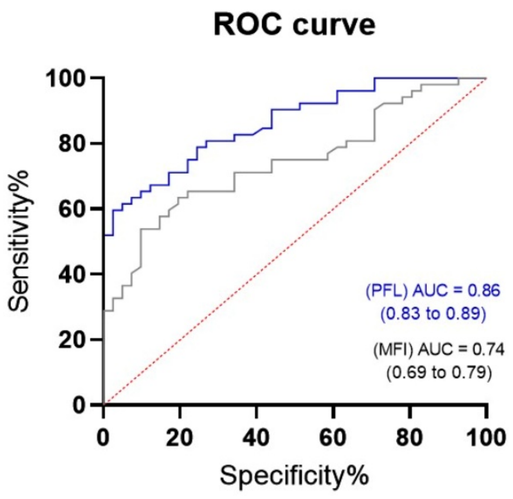

Figure S3. Receiver operating characteristic (ROC) curve generated to assess the performance of the classification model in discriminating between individuals with and without malaria. The area under the ROC curve (AUC) for each biomarker was calculated, accompanied by a 95% confidence interval to assess the estimate's accuracy. Values closer to 1 indicate better discrimination between classes. ROC curves were compared using the DeLong test, which evaluates the difference between the AUCs of two models considering a significance level of 0.05.

Table S1. Results of the two flow cytometry analysis methods using latex beads coupled with anti-*P. vivax* lactate dehydrogenase (pLDH) polyclonal antibodies.

Method	Sensitivity (%)	Specificity (%)	PPV (%)	NPV (%)
PFL	64	97	97	57
MFI	53	89	95	33

PFL: Percentage of fluorescent labeling; MFI: mean fluorescence intensity; PPV: Positive prediction value; NPV: Negative prediction value.
